# miR-21 and miR-145 cooperation in regulation of colon cancer stem cells

**DOI:** 10.1186/s12943-015-0372-7

**Published:** 2015-05-01

**Authors:** Yingjie Yu, Pratima Nangia-Makker, Lulu Farhana, Sindhu G. Rajendra, Edi Levi, Adhip PN Majumdar

**Affiliations:** Department of Veterans Affairs Medical Center, 4646 John R, Detroit, MI 48201 USA; Karmanos Cancer Center, Detroit, MI 48201 USA; Departments of Internal Medicine, Wayne State University, Detroit, MI 48201 USA

**Keywords:** Colorectal cancer, Negative feedback, Chemo-resistance

## Abstract

**Background:**

Acquired drug resistance is one of the major reasons for failing cancer therapies. Although the reasons are not fully understood, they may be related to the presence of cancer stem cells (CSCs). We have reported that chemo-resistant (CR) colon cancer cells, highly enriched in CSCs, exhibit a marked up-regulation of miR-21 and that down-regulation of this miR renders the CR cells more susceptible to therapeutic regimens. However, the underlying molecular mechanism is poorly understood. The aim of this investigation is to unravel this mechanism.

**Methods:**

The levels of miR-145 and miR-21 were manipulated by transfection of mature, antago-miRs or pCMV/miR-145 expression plasmid. Quantitative RT-PCR or/and Western blots were performed to examine the expression of CD44, β-catenin, Sox-2, PDCD4, CK-20 and k-Ras. Colonosphere formation and SCID mice xenograft studies were performed to evaluate the tumorigenic properties of CSC-enriched colon CR cells.

**Results:**

We investigated the role that microRNAs (miRs), specifically miR-21 and miR-145 play in regulating colon CSCs. We found the expression of miR-21 to be greatly increased and miR-145 decreased in CR colon cancer cells that are highly enriched in CSC, indicating a role for these miRNAs in regulating CSCs. In support of this, we found that whereas forced expression of miR-145 in colon cancer cells greatly inhibits CSCs and tumor growth, up-regulation of miR-21 causes an opposite phenomenon. In addition, administration of mature miR-145 or antagomir-21 (anti-sense miR-21) greatly suppresses the growth of colon cancer cell xenografts in SCID mice. This was associated with decreased expression of CD44, β-catenin, Sox-2 and induction of CK-20 indicating that administration of miR-145 or antagomir-21 decreases CSC proliferation and induces differentiation. *In vitro* studies further demonstrate that miR-21 negatively regulates miR-145 and *vice versa.* k-Ras appears to play critical role in regulation of this process, as evidenced by the fact that the absence of k-Ras in CR colon cancer cells increases miR-145 expression, suppresses miR-21, and interrupts the negative cooperation between miR-21 and miR-145.

**Conclusions:**

Our current observations suggest that miR-21, miR-145, and their networks play critical roles in regulating CSCs growth and/or differentiation in the colon cancer and progression of chemo-resistance.

## Background

Despite advances in medicine, nearly 50% of patients with colorectal cancer (CRC) show tumor recurrence, for which the outcome remains poor; the median survival following recurrence is only 13.3 months [1]. The higher recurrence of CRC is thought to be the result of drug-resistance of cancer cells. Although the reasons for drug resistance are not fully understood, they may be related to the presence of cancer stem or cancer stem-like cells (CSCs/CSLCs) that are thought to play pivotal role in tumor initiation, progression, metastasis, and its relapse [2-4].

CSCs/CSLCs are a small population of self-renewing undifferentiated cells within a tumor that have been shown to be resistant to radiation and chemotherapy [2]. CSCs/CSLCs isolated from different solid tumors, including the colon are usually identified by specific surface epitopes. Colon CSCs/CSLCs have been shown to express CD44, CD166, CD133 and ESA (epithelial-specific antigen, also known as EpCAM) surface markers [5]. We have observed that in humans, colon CSCs/CSLCs are present not only in premalignant adenomas but also in normal appearing colonic mucosa and that the population of CSCs/CSLCs increases with advancing age, suggesting that they may be partly responsible for the age-related rise in colorectal cancer [6].

The standard therapy for advanced CRC includes surgery followed by chemotherapy or other effective therapeutic regimen to eliminate any remaining cancer cells. 5-Fluorouracil (5-FU) based regimen such as FOLFOX (5-FU plus Oxaliplatin and Folinic acid) remains the backbone of colorectal cancer chemotherapeutics but with limited success. Near 50% tumor recurrence rate suggests that the cells survive chemotherapy and may lead to cancer recurrence. We have reported that 5-Fluorouracil and Oxaliplatin (FU-Ox) resistant [chemo-resistant (CR)] colon cancer HCT116 and HT29 cells exhibit enrichment of CSCs/CSLCs, elevated levels of mature miR-21 and that miR-21 induces stemness in colon cancer cells [7,8].

MicroRNAs (miRNAs) comprise a broad class of small (19–22 nucleotide) endogenous RNAs that negatively control the expression of the target genes by cleaving mRNA or through translation repression [9], and can function as oncogenes or tumor suppressors depending on the target. More than 1500 human miRNAs are annotated in the miRBase and many of them are aberrantly expressed in several pathological conditions, including cancer. In colorectal cancer, miR-21 has been reported to function as an oncomiR (a miRNA with oncogenic properties) due to its key role in several processes of tumor promotion, invasion and metastasis [10,11]. We found miR-21 to induce stemness of colon cancer cells [8]. Furthermore, overexpression of miR-21 has been shown to dramatically reduce the therapeutic efficacy of 5-FU [12]. Although the underlying mechanisms for regulation of miR-21 in CRC remain to be defined, we reported EGFR inhibitor Cetuximab (mAb to EGFR) to decrease its expression suggesting a role for EGFR in regulating miR-21[13]. In contrast to miR-21, miR-145 is a p53 regulated tumor suppressor, whose down-regulation has been found in colorectal and other cancers [14,15]. It regulates stem cell renewal and pluripotency by suppressing multiple pluripotent genes: OCT4, SOX2 and KLF4 [14].

Herein, we report that stable over expression of miR-145 in colon cancer HCT-116 or HT-29 cells or in the corresponding CR colon cancer cells significantly induces differentiation and inhibits their growth in vitro. A similar phenomenon occurs following down-regulation of miR-21 in CR colon cancer cells. The tumorigenic potential of parental and CR-HT-29 cells in SCID mice was inhibited by administration of miR-145 or anti-miR-21. In addition, we observed downregulation of pluripotency factors Oct4, Sox2, Nanog as well as miR-21 following overexpression of miR-145 in colon cancer cells. We also report that miR-21 negatively regulates miR-145 and *vice versa.* k-Ras appears to play critical role in the regulation of this process, as evidenced by the fact that the absence of k-Ras in CR colon cancer cells increases miR-145 expression, suppresses miR-21, and interrupts the cooperation between miR-21 and miR-145.

## Results

### Over-expression of miR-145 induces differentiation, inhibits stemness and xenograft tumors in SCID mice

More than 80% of colorectal cancers arise from adenomatous polyps that are known to contain CSCs/CSLCs [6] and dysregulation of miRNAs [16]. We have reported that the expression of miR-21 is greatly increased in chemo-resistant (CR) colon cancer cells that are highly enriched in CSC, and forced expression of miR-21 in colon cancer cells greatly increases CSC population accompanied by induction of tumor growth, indicating miR-21 regulates stemness of colon cancer cells [8,17].

**Figure 1:**
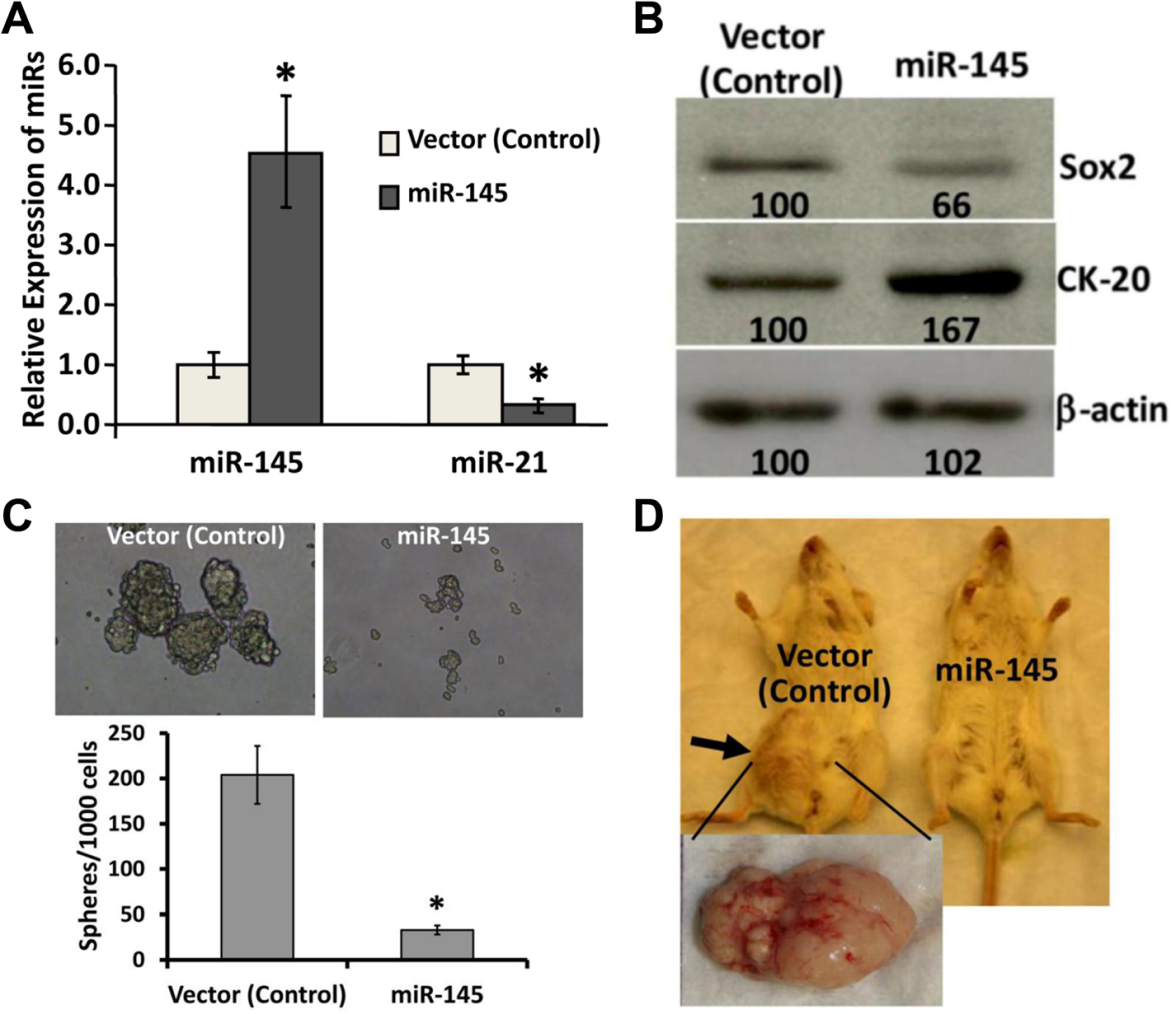
Over-expression of miR-145 in colon cancer HCT-116 cells by stably transfected pCMV/miR-145 downregulates miR-21 and induces differentiation, inhibits stemness and produces no visible tumors in SCID mice. **(A)** qRT-PCR showing up-regulation of mature miR-145 and down regulation of miR-21 in HCT-116 cells that were stably transfected with pCMV/miR-145 plasmid or the corresponding vector, *P < 0.001. **(B)** Western-blot showing decreased expression of Sox2 and increased expression of CK-20 in miR-145 over-expressing HCT-116 cells, compared to the corresponding vector-transfected control cells. β-actin was used as a loading control, **(C)** Representative photographs showing colonospheres formed by the cells from miR-145 expressing clones, derived from HCT-116 cells, stably transfected with pCMV-miR-145 plasmid or the corresponding vector (top panel), histogram showing the number of colonspheres formed by miR-145 over-expressing HCT-116 cells, compared to the corresponding vector-transfected control cells (bottom panel, *P < 0.001), **(D)** while administration of miR-145 over-expressing HCT-116 cells produces no visible tumors in SCID mice, the vector-transfected HCT-116 cells (control) induces large tumors in SCID mice. The data represent means of three independent experiments.

To determine the putative functional properties of miR-145 in the development of colorectal tumor and its relation to miR21 expression, pCMV/miR145 plasmid (Origene, Rockville, MD) was stably transfected in HCT-116 cells. As determined by qRT-PCR (real time PCR) analysis, the expression of miR-145 was found to be 4-fold higher in the miR-145 positive cells, compared to empty vector (Figure 1A). In contrast, miR-21 was decreased by 50% in miR-145 overexpressing cells, compared to the vector-transfected control cells (Figure 1A). Western blot analysis revealed that the levels of Sox2, the target of miR-145 were decreased by 34% and the expression of cytokeratin-20 (CK-20), the differentiation marker was increased by 67%, compared to the vector-transfected controls (Figure 1B).

The next set of experiments was carried out to determine whether miR-145 may regulate stemness of colon cancer cells. The functional property of cancer stem cells is defined by their ability to form sphere/spheroid (*in vitro*) in serum-free medium containing growth factors (stem cell medium) when plated in ultra low-attachment plates under extreme limited dilutions [18]. To determine whether and to what extent miR-145 over-expression affects the sphere forming properties of colon cancer cells, HCT-116 cells stably transfected with pCMV/miR-145 or pCMV vector (control) were subjected to an extreme limiting dilution analysis (ELDA). Number of spheroids formed as well as the frequency to form spheroids by miR-145 expressing HCT-116 cells was found to be about 88% lower than those formed by the vector-transfected control cells (p <0.001) (Figure 1C and Table 1). Further, the average diameter of spheroids, obtained from miR-145 overexpressing cells, was found to be smaller than the controls (Table 1 and Figure 1C). Tumorigenic potential of the miR-145 overexpressing cells was analyzed by generating xenograft tumors in SCID mice. We observed that while the HCT-116 pCMV empty vector-transfected (control) cells formed palpable tumor within 3 weeks of injection, no tumor could be detected with HCT-116 pCMV/miR-145 cells even after 6 weeks (Figure 1D).

**Table 1:** **Sphere-forming frequency, diameter and/or self-renewal ability of cells from colonospheres of stably transfected with pCMV (Vector), pCMV/miR-145 (miR-145) colon cancer HCT-116 cells**

	**Vector**	**miR-145**	***P*** **value**
Sphere-forming frequency (95% CI)	1/11 (1/17-1/4)	1/88 (1/196-1/40)	<0.001
Diameter (μm)	104.0 ± 18.5	47.0 ± 13.2	<0.001
Self-renewal (spheres per 200 cells)	13 ± 3	-	<0.001

Data are pooled from three independent experiments for each. CI, confidence interval.

### miR-145 and miR-21 cooperation plays a role in regulating cancer stem cell proliferation and differentiation

**Figure 2:**
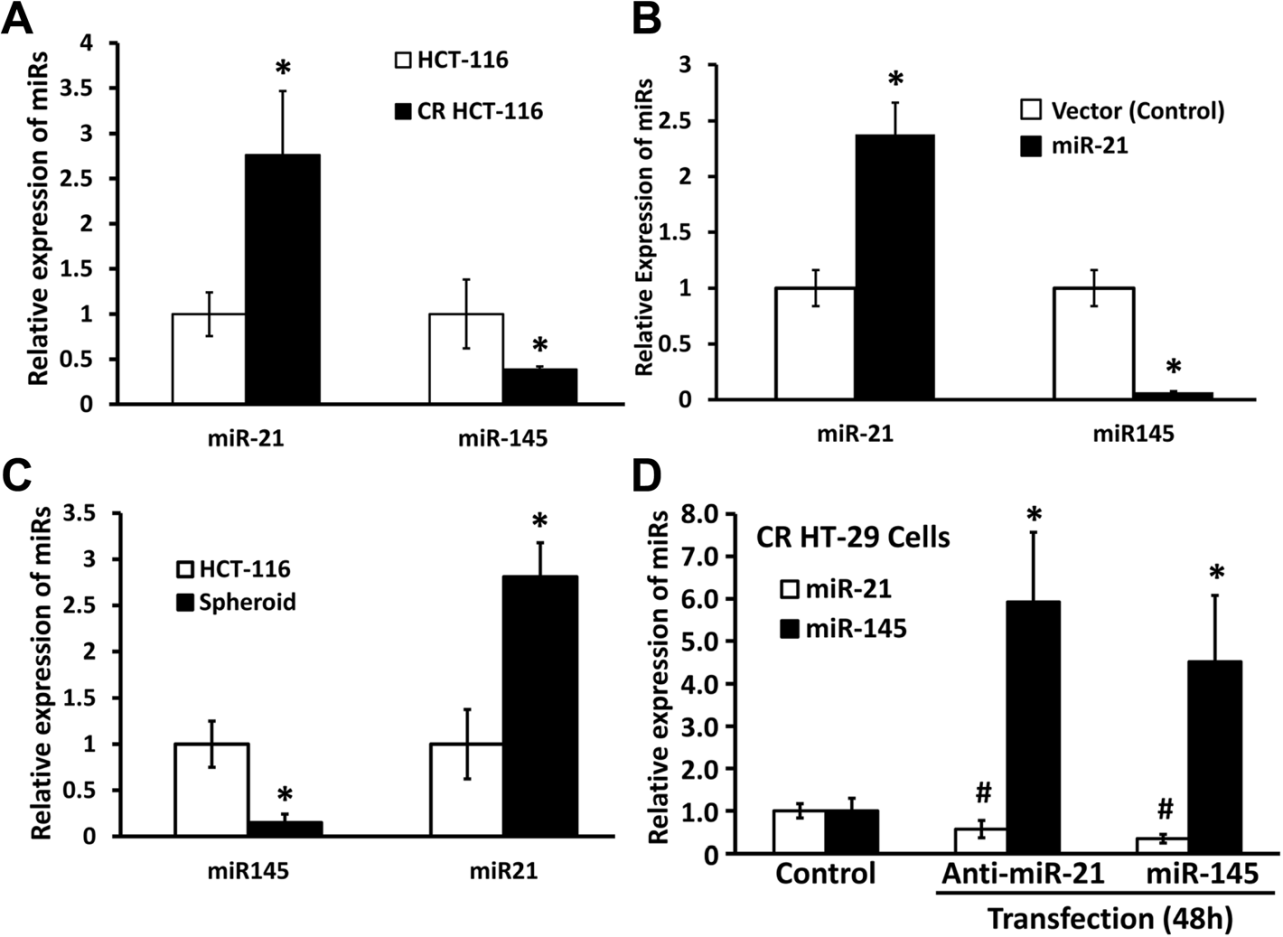
Chemo-resistance, overexpression of miR-21 and colonosphere formation in colon cancer HCT-116 cells, all of which are associated with increased miR-21 levels, lead to down-regulation of miR-145, and miR-145 and miR-21 negatively regulate each other in chemo-resistant (CR) colon cancer cells. **(A)** qRT-PCR showing up-regulation of mature miR-21 and down-regulation of mature miR-145 in CR HCT-116 cells when compared with the parental HCT-116 cells. **(B)** qRT-PCR showing over-expression of miR-21 (stably transfected cells) leads to decreased miR-145 compared to the vector-transfected control. **(C)** qRT-PCR showing down-regulation of miR-145 and up-regulation of mature miR-21 in colonospheres formed by parental HCT-116 cells. **(D)** qRT-PCR showing relative expression of miR-21 and miR-145 in CR HT-29 cells at 48 h following transfection of mature miR-145 or anti-miR-21. All the data represent means ± SD of three independent experiments, *P < 0.001, compared to the control.

In the next set of experiments we tested whether there is a cooperation between miR-145 and miR-21 and how this cooperation may regulate cancer stem cell proliferation and differentiation. To conduct this experiment, we utilized CR colon cancer cells that are enriched in CSCs and exhibit a 3–5 fold increase in the precursor and mature miR-21 as shown in Figure 2A, an observation similar to what we reported earlier [8,17]. We observed that the expression of tumor suppressive miR-145 in CR HCT-116 cells was decreased by 62% and in miR-21-overexpressing HCT-116 cells by 90%, when compared with their corresponding parental or empty vector control cells (Figure 2A and B). In addition, we observed that while the levels of miR-145 were decreased by ~70% in colonospheres formed by the parental HCT-116 cells, the expression of miR-21 was increased by ~180% in these colonospheres, when compared with the corresponding parental HCT-116 cells (Figure 2C). These results indicate that overespression of miR-21 is associated with downregulation of miR-145 and vice versa in colon cancer cells and that both miR-21 and miR-145 are involved in regulating the growth of colonospheres, enriched in CSCs.

To further investigate the cooperation between miR-145 and miR-21 in CR colon cancer cells, we regulated the levels of miR-145 (increased) or miR-21(decreased) by transfecting mature miR-145 or anti-miR-21 in CR-HT-29 cells in vitro. As expected, miR-145 was increased and miR-21 was decreased after transfection of miR-145 and anti-miR-21, compared with corresponding control in CR colon cancer cells (Figure 2D). Following transfection of anti-miR21 in CR-HT-29 cells, there was a marked 6-fold increase of miR-145 (Figure 2D). On the other hand, in miR145-transfected CR HT-29 cells, the levels of miR21 were decreased by 70%, compared to the corresponding control (Figure 2D). Collectively, the current data suggest a negative feedback between the tumor suppressor miR-145 and oncomiR miR-21 in CR colon cancer cells.

### miR-145 and, anti-miR-21 inhibit tumorigenic potential of chemo-resistant (CR) colon cancer HT-29 cells in SCID mice

**Figure 3:**
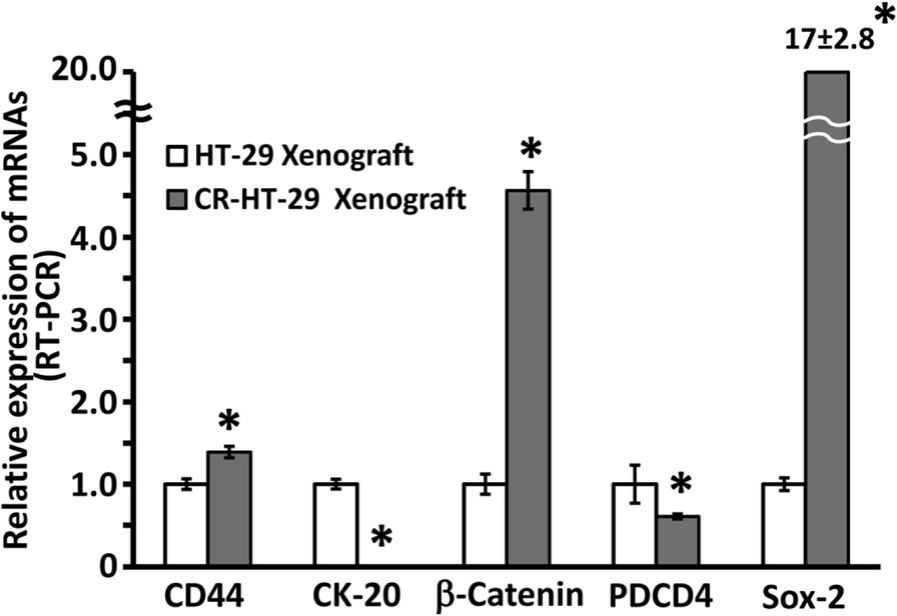
Presence of markers of CSC and their growth and differentiation in xenografts derived from parental HT-29 or CR HT-29 cells. qRT-PCR showing relative expression of CD44, CK20, β-Catenin, PDCD4 and Sox2 in xenografts derived from parental HT-29 or CR HT-29 cells. The data represent means ± SD of three independent experiments, *P < 0.001, compared to the control.

To determine whether and to what extent miR-145 or anti-miR-21 would affect the tumorigenic potential, xenografts in SCID mice formed by colon cancer parental HT-29 or CR-HT-29 cells were first analyzed for the presence of markers of CSCs/CSLCs and their growth and differentiation. We observed that the expression of CD44 (colon CSC marker), β-catenin (stem cell growth regulator) and SOX2 (a miR-145 target) was 39%, 356% and 1600% higher, respectively, whereas the levels of PDCD4 and CK20 were 40% and 95% lower in CR HT-29 xenografts, when compared with the values from xenografts of parental HT-29 cells (Figure 3).

**Figure 4:**
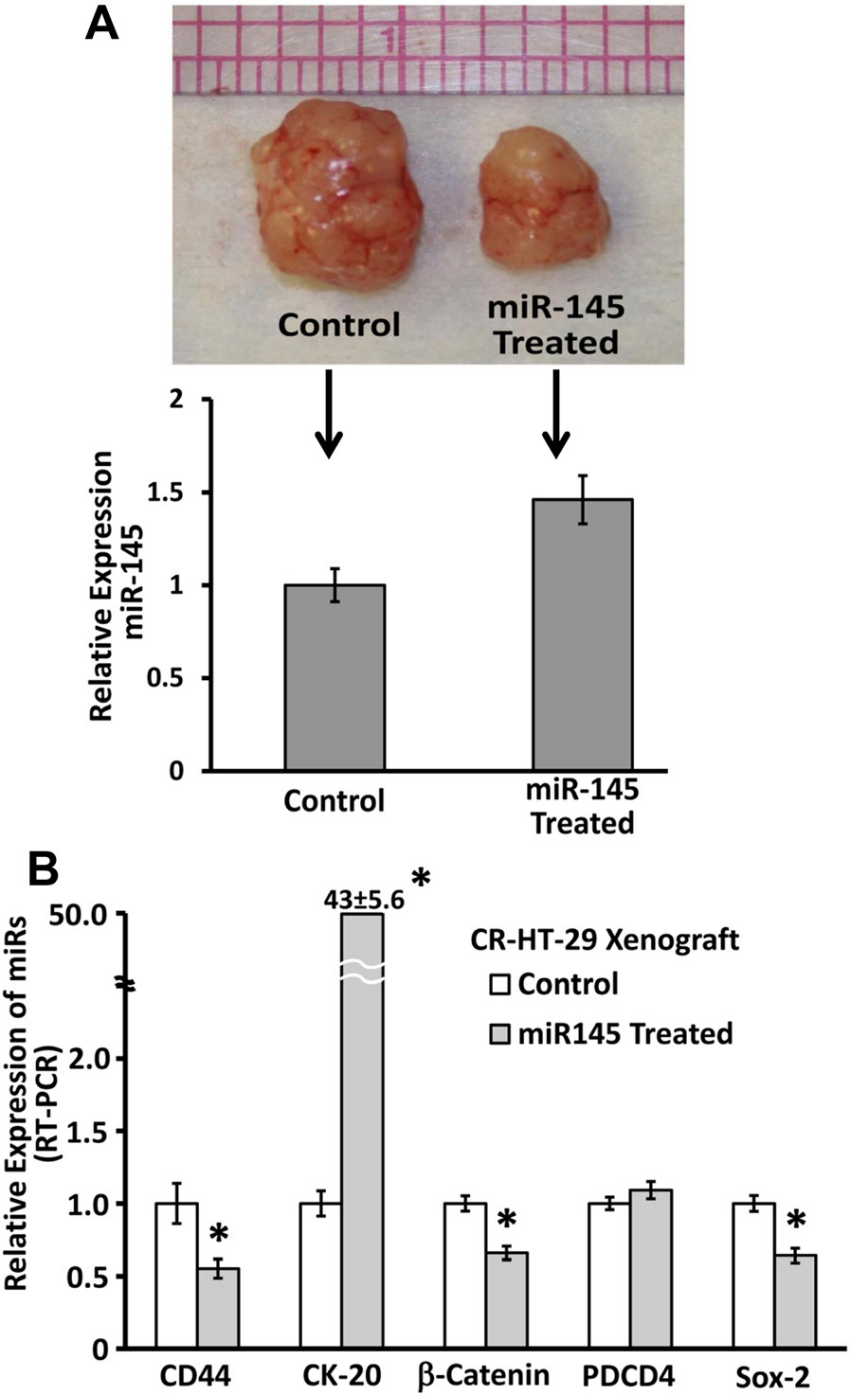
miR-145 reduces tumorigenic potential of chemo-resistant (CR) colon cancer HT-29 cells in SCID mice. **(A)** administration of PEI/miR-145 complexes suppresses xenograft growth in SCID mice. CR HT-29 cells (2.5 × 10^5^ cells) were injected with 50% Matrigel. Once palpable tumors developed (~3 weeks), the treatment (i.p.; weekly) was initiated. Upper panel shows intact xenograft tumor after treatment with PEI/miR-145 or the corresponding controls, and the lower panel shows the relative levels of miR-145 in the tumor after treatment with PEI/miR-145 or the corresponding controls. **(B)** qRT-PCR showing relative expression of CD44, CK20, β-Catenin, PDCD4 and Sox2 in tumors derived from CR HT-29 cells after treatment with PEI/miR-145 (miR145 Treated ) or the corresponding controls (Control). The data represent means ± SD of three independent experiments, *P < 0.001, compared to the control.

We then determined the effectiveness of miR-145 in suppressing the growth of colon cancer xenografts in SCID mice. HCT-116 cells have consistently active mutated k-Ras, while HT-29 cells harbor normal k-Ras. Additionally, endogenous miR-145 is highly repressed in HT-29 cells, therefore, in this investigation, 2.5 × 10^5^ CR HT-29 cells were injected with 50% Matrigel in each mouse. Once palpable tumors were developed (~3 weeks), Polyethylenimines (PEI)/miRNA-145 complex treatment (i.p.; weekly) was initiated. The results revealed that treatment with (PEI)/miRNA-145 complex significantly suppressed tumor growth, compared with the vehicle-treated controls (Figure 4A). The relative level of miR-145 in the tumor of SCID mice injected with miR-145 was about 50% higher than the control tumor (Figure 4A; lower panel).

At the end of the experiment, a small portion of the tumors from CR-HT-29 cells and those treated with (PEI)/miRNA-145 or vehicle complex was used for RNA extraction and analyzed for the expression of various markers of CSC growth and differentiation by real time PCR. The expression of CD44, β-catenin and SOX2 was decreased by 45%, 34% and 36%, and PDCD4 and CK20 was increased significantly by 10% and over 4000%, respectively, in CR-HT-29 xenografts from (PEI)/miRNA-145 complex-treated xenograft, compared to those fromed by vehicle- treated (control) CR HT-29 cells (Figure 4B). These results indicate that administration of miR-145 greatly decreases CSC proliferation and induces differentiation leading to suppression of tumor growth in SCID mice.

## Discussion

Although many factors may contribute to colorectal cancer, chemo-resistance and relapse of cancer and metastasis, it is reasonable to speculate that CSCs play critical role in these processes. Our current data further suggest that the tumor suppressor miR-145, oncomiR miR-21 and their networks are critically involved in regulating these events.

Earlier, we reported that exposure of colon cancer HCT-116 or HT-29 cells to the combination of 5-Fluorouracil (5-FU) and Oxaliplatin (Ox), the backbone of colorectal cancer chemotherapy, inhibited their growth and led to enrichment of CSC/CSLC phenotype, where the levels of miR-21 were greatly increased indicating a potential role for this miR in regulating CSCs/CSLCs [7,8]. In addition, we reported miR-21 to induce stemness of colon cancer cells by down-regulating TGFβR2 and that down-regulation of miR-21 induces differentiation of CR colon cancer cells and enhances susceptibility to therapeutic regimens [8,17].

We also demonstrated that miR-21 regulates CSC function as well as its growth and differentiation by modulating its direct targets such as TGFβR2, PDCD4 and PTEN, as reported earlier [8,17,19]. Our current data show that miR-21 also regulates the expression of the tumor suppressor miR-145. The latter is supported by the following observations: (a) miR-21 is up-regulated, whereas miR-145 is down-regulated in colon cancer CR-HCT-116 cells, highly enriched in CSCs/CSLCs; (b) forced expression of miR-21 through transfection of mature miR-21 in colon cancer HCT-116 cells decreases the expression of miR-145; (c) knock-down of miR-21 by anti-miR-21 increases miR-145 in CR colon cancer cells. These results indicate that miR-21 negatively regulates the expression of miR-145 in colon cancer cells. On the other hand, miR-21 can be decreased by elevating the levels of miR-145 through transfection of mature miR-145 in parental and CR colon cancer cells. These data further suggest that a negative feedback exists between miR-21 and miR-145.

miR-145 is a p53-regulated gene. p53 can induce its transcription and enhance the post-transcriptional maturation of miR-143/miR-145 cluster [15,20] in response to DNA damage by interacting with the Drosha processing complex [21]. Activated Ras can suppress miR-143/145 cluster transcription through Ras-responsive element-binding protein (RREB1), which represses the miR-143/145 promoter [22]. Hatley and colleagues have reported that miR-21 increases RAS signaling activity and therefore leads to repression of the miR-143/145 cluster [23]. These reports support our data and further demonstrate the mechanism by which miR-21 negatively regulates tumor suppressor miR-145.

Down-regulation of miR-145 has been found in multiple tumors including colon, breast, prostate, pancreas etc. [14,15]. In fact, miR-145 has been well documented as a tumor suppressor gene because it negatively regulates multiple oncogenes such as Myc, K-Ras, IRS-1, ERK5 [15,24]. Moreover, miR-145 negatively regulates junctional cell adhesion molecule (JAM-A), fascin and MUC1 and suppresses breast cancer cell motility and invasiveness [25,26]. miR-145 also inhibits colon cancer cells’ proliferation and sensitizes them to 5-fluorouracil by targeting oncogenic FLI1 [27].

miR-21 expression is regulated at multiple levels, including transcription and post-transcriptional processing. Talotta et al. [28] have reported that the miR-21 is induced by AP1 in response to Ras. They have also demonstrated that PDCD4, a pro-apoptotic gene and the target of miR-21, is a negative regulator of AP1. The miR-21-mediated down-regulation of PDCD4 is essential for the maximal induction of AP1 activity in response to Ras. These data reveal a mechanism of positive auto-regulation of the AP1 complex in Ras transformation and disclose the function of oncomiRs as critical targets and regulators of AP1 in tumorigenesis [29]. Kern et al. [30] also showed that EGF/Ras efficiently induced the miR-21 primary transcript, but this does not rapidly and simply translate into higher mature miR-21 levels.

**Figure 5:**
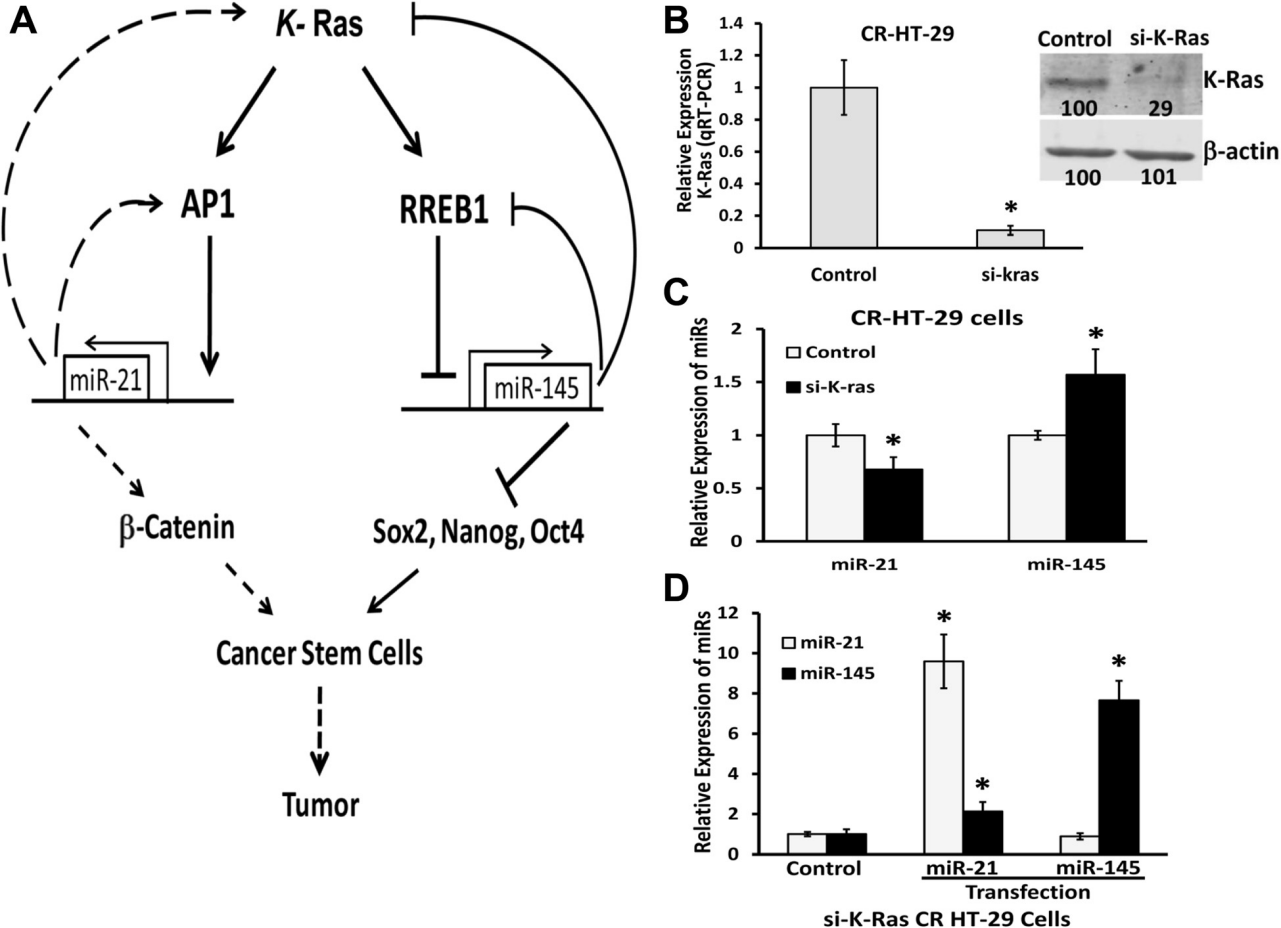
The feedback circuits between miR-21 and miR-145. **(A)** Schematic representation of circuits between miR-21 and miR-145, and themselves in cancer stem cells. Herein, it is suggested that miR-21 represses miR-145 transcription by stimulating Ras activity that causes RREB1 to repress miR-145 transcription, and that miR-145 inhibits miR-21 transcription through knockdown target Ras (*K*-RAS) and decreased AP1, the main transcription factor of miR-21. In the miR-21 positive feedback loop, miR-21 is auto-upregulated via indirect upregulation of AP1. In the miR-145 positive feedback loop, miR145 increases its transcription via knock-down Ras and RREB1, the transcription repressor of miR-145. Pleuripotency factors Sox2, Nanog and Oct4 are the direct targets of miR-145. **(B)** Real-time qRT-PCR and Western blot analysis showing down-regulation of k-Ras following transfection with k-Ras siRNA for 48 h, compared to the corresponding scrambled siRNA transfected control cells. β-actin was used as a loading control. **(C)** qRT-PCR showing down-regulation of k-Ras in CR HT-29 cells produces reduction in miR-21 and increases in miR-145 expression, when compared with the corresponding controls **(D)** qRT-PCR showing relative expression of miR-21 and miR-145 in k-ras-downregulated CR HT-29 cells at 48 h following transfection of mature miR-145 or miR-21. All the qRT-PCR data represent means ± SD of three independent experiments; *P < 0.001, compared to the control.

In view of these reports together with our current observations prompted us to hypothesize that the negative feedback between miR-21 and miR-145 mediated by Ras signaling pathway plays a crucial role in the induction of CSC proliferation or/and differentiation in CR colon cancer cells, as depicted in Figure 5A. Restoration of miRs level by increasing miR-145 or decreasing miR-21 can dislodge Ras mediated feedback between miR-145 and miR-21 and inhibit tumor growth. Indeed, this hypothesis has been supported by our *in vitro* (Figure 5B-D) and *in vivo* experiments (Figure 4). In the *in vitro* studies, downregulation of k-Ras in CR colon cancer cells was achieved by transfection with corresponding siRNA and the controls with scrambled siRNA. The protein as well as mRNA levels of k-Ras were substantially reduced (70–80% reduction), as determined by Western blot and qRT-PCR analyses, when compared with the corresponding controls (Figure 5*B*). Down-regulation of k-Ras in CR HT-29 cells, that caused a 45% increase in miR-145, produced a 35% reduction in miR-21, when compared with the corresponding controls (Figure 5C). Forced expression of miR-21 in k-Ras downregulated CR-HT-29 cells resulted in 1-fold increase in miR-145 (Figure 5D). But, increasing the levels of miR-145 in k-ras downregulated CR-HT-29 cells resulted in no significant reduction in miR-21, compared with corresponding control (Figure 5D). These observations are in contrast to those depicted in Figure 2D, where are show that in cell with intact k-Ras, downregulation of miR-21 produced 6-fold augmentation of miR-145, forced expression of miR-145 resulted in 70% reduction in miR-21. These observations suggest that k-Ras mediates the negative feedback between the tumor suppressor miR-145 and oncomiR miR-21 in CR colon cancer cells. This inference is further supported by our *in vivo* experiments which demonstrate that increase of miR-145 or inhibition of miR-21 by injecting PEI mediated chemically engineered modified single-stranded RNA or analogues complementary to miRNA which are efficient, specific and long-lasting replacements or silencers of endogenous miRNAs in mice [24,31] leads to suppression of xenograft growth in SCID mice. The growth inhibition was associated with decreased proliferation of CSCs and induction of their differentiation, as evidenced by reduction in expression of CD44 and induction of CK20 following upregulation of miR-145 or down-regulation of miR-21.

## Conclusions

Our current observations suggest that dysregulation of miR-21 and miR-145 plays a central role in the growth of CSCs in chemo-resistant colon tumors by regulating a network of genes that are critically involved in tumor progression, metastasis, and relapse of colorectal cancer.

## Materials and methods

### Cell lines and cell cultures

Human colon cancer HCT-116 and HT-29 cells were obtained from the American Type Culture Collection (ATCC, Rockville, MD). They were expanded and frozen in aliquots. Fresh aliquots were used every 6–7 months. The cells were maintained in Dulbecco's modified Eagle medium (DMEM; 4.5 g/L d-glucose) supplemented with 10% FBS (Invitrogen, Grand Island, NY) and 1X antibiotic/antimycotic (Invitrogen, Grand Island, NY) in tissue culture flasks, 5-Fluorouracil and Oxaliplatin (FuOx) resistant [chemo-resistant (CR)] colon cancer HCT116 and HT29 cells were generated as described earlier [7,8] in our laboratory and were maintained in normal culture medium containing 2× FuOx (50 μM 5-Fu + 1.25 μM Ox) in tissue culture flasks in a humidified incubator at 37°C in an atmosphere of 95% air and 5% carbon dioxide. The medium was changed two times a week, and cells were passaged using 0.05% trypsin/EDTA (Invitrogen, Grand Island, NY).

### Western blot analysis

Western blot analysis was performed according to our standard protocol [32,33]. Briefly, the cells were solubilized in lysis buffer and the protein concentration was determined by the Bio-Rad Protein Assay kit (Bio-Rad, Hercules, CA). The proteins were separated by SDS-PAGE and transferred to PVDF membranes (Millipore). The membranes were incubated overnight at 4°C with primary antibodies after blocking. The membranes were subsequently washed and incubated with appropriate secondary antibodies. The protein bands were visualized by enhanced chemiluminescence (ECL) detection system (Amersham, Piscataway, NJ). Whenever appropriate, the membranes were stripped and re-probed with β-actin for verification of protein loading.

### Transfection of miR-145, miR-21, anti-miR-21 or k-Ras siRNA in colon cancer cells

For transfection of miR-21/145, anti-miR-21 or/and siRNA in the CR-HCT-116 or CR-HT-29 cells, Lipofectamine 2000 transfection reagent (Invitrogen Corp.) and serum-free Opti-MEM (Invitrogen Corp.) medium were prepared according to the manufacturer's instructions. Briefly, the cells were plated in six-well tissue culture plates with normal growth medium and incubated overnight to achieve 40–60% confluence. Next day, the medium was removed, washed with serum-free Opti-MEM (Invitrogen Corp.) medium prior to adding the complexes containing 100 pmol of scrambled (control), k-Ras siRNA (Integrated DNA Technologies, Coralville, IA) or/and pre-miR-145, pre-miR-21. After 2 days of transfection, the cells were collected and analyzed for protein expression of k-Ras using western blot and for quantitation of miRNA-21 and miR-145 by qRT-PCR according to our standard protocol.

### Isolation of RNA and quantitative polymerase chain reaction analysis

Total RNA was extracted from different cells using RNA-STAT solution (Tel Test, Friendswood, TX) according to the manufacturer's instructions. The total RNA was treated with DNase I and purified with phenol-chloroform. RNA concentration was measured using a NanoDrop 2000C spectrophotometer.

Quantitative reverse transcription-polymerase chain reaction (qRT-PCR) was performed using the GeneAmp RNA PCR Kit (Applied Biosystems, Foster City, CA). 5 μl of cDNA products were amplified with SYBR Green Quantitative PCR Master Mix (Applied Biosystems). PCR primers used were as follows: CD44, forward: 5′-aaggtggagcaaacacaacc-3′, reverse: 5′-actgcaatgcaaactgcaag-3′; CK-20, forward: 5′-tgaagagctgcgaagtcaga-3′ and reverse: 5′-gaagtcctcagcagccagtt-3′, β-Catenin forward: 5'-ggaaggtctccttgggactc-3' and reverse: 5'-ataccacccacttggcagac-3'; PDCD4, forward: 5'-ggtgggccagtttattgcta-3' and reverse: 5'-cggtacccttatccagagca-3'; Sox2, forward: 5'-aaccccaagatgcacaactc-3' and reverse: 5'-gcttagcctcgtcgatgaac-3'; k-Ras, forward: 5'-tgtggtagttggagctggtg-3' and reverse: 5'-tccaagagacaggtttctcca-3'; β-actin forward: 5′-cccagcacaatgaagatcaa-3′ and reverse 5′-acatctgctggaaggtggac-3′. Reactions were carried out in Applied Biosystems 7500 Real-Time PCR System; The running conditions for PCR were as follows: for activating the DNA polymerase, hot start was performed for 10 min at 95°C, and then cycling at 95°C for 15 s and 60°C for 1 min for a total of 40 cycles.

### Quantitation of miRNA-21 and miR-145

TaqMan microRNA assays were used to quantitate miR-21 and miR-145 in different colon cancer cells according to the manufacturer’s instructions (Applied Biosystems, Foster City, CA). Briefly, cDNA synthesis was carried out with the TaqMan MicroRNA reverse transcription kit (Applied Biosystems). The miRNA reverse transcription-PCR (RT-PCR) primers for *miR-21*, miR-145 and endogenous control RNU6B were purchased from Applied Biosystems. Real-time quantitative RT-PCR (qRT-PCR) analysis was carried out using Applied Biosystems 7500 Real-time PCR System. The PCR mix containing TaqMan 2× Universal PCR Master Mix were processed as follows: 95°C for 10 min and then 95°C for 15 s, 60°C for 60 s for up to 40 cycles. Signal was collected at the endpoint of every cycle. The gene expression Δ*C*_T_ values of miRNAs from each sample were calculated by normalizing with internal control RNU6B and relative quantitation values were plotted.

### Generation of miR-145 over-expressing HCT-116 cells

pCMV-miR-145 plasmid carrying pre-microRNA-145 and 250–300 nts up and down-stream flanking sequence (Origene, Nockville, MD) or empty vector DNA alone (pCMV) was transfected into HCT-116 cells by Lipofectamine™ 2000 reagent according to the manufacturer's instructions (Invitrogen Corp, CA). Several independent sublines (colonies) were generated over 8–10 wk of the selection period in the presence of 0.6 mg/ml G418 (Neomycin). Colonies were picked at random and grown as individual cell lines in the presence of 0.4 mg/ml G418. Each cell line was subjected to RT-PCR analysis to evaluate miR-145 expression.

### Formation of colonospheres and Extreme limiting dilution analysis

The ability of miR-145-overexpressing and parental HCT-116 cells to form spheres in suspension was evaluated as described previously [18]. Briefly, primary colonospheres were generated by incubating the limited number of pCMV and miR-145 stably transfected HCT-116 cells at a concentration of 1000 cells per 500 μL in serum-free stem cell medium (SCM) containing DMEM/F12 (1:1) supplemented with B27 (Life Technologies, Gaithersburg, MD), 20 ng/ml EGF (Sigma, St Louis, MO), 10 ng/ml fibroblast growth factor (Sigma), and antibiotic-anti-mycotic in 24-well plates (Corning Inc, Lowell, MA) for 10 days. The formed colonospheres were centrifuged (1000 rpm), dissociated with 0.05% trypsin/EDTA and reseeded in SCM. The single-cell suspension derived from colonospheres that have undergone 15 or more serial passages were used for all experiments.

Self-renewing/regeneration abilities of the spheres, derived from pCMV and miR-145 stably transfected HCT-116 cells, were analyzed for secondary colonospheres formation in the following manner. Primary colonospheres formed over a period of ten days in SCM containing DMEM/F12 (1:1) were collected by centrifugation, dissociated with 0.05% trypsin/EDTA, and subsequently passed through a 40 μM sieve to obtain single cell suspensions. An equal number of cells from primary colonospheres culture were plated (100 cells/500 μL in SCM) in ultra low-attachment wells. The secondary colonospheres formed after five days were recorded for their number and size by light microscopy.

Extreme limiting dilution analysis (ELDA) was performed essentially according to Hu and Smyth with slight modifications as described by us [18,34]. Briefly, single cell suspension obtained from adherent miR-145-expressing HCT-116 and vector-transfected (control) HCT-116 cells were plated at concentrations of 200, 20 and 2 cells per 100 μl SCM (24 well for each dilution) in 96-well plates and incubated for 5 days. At the end of 5 days, the number of wells showing formation of colonospheres was counted. The frequency of sphere formation in a particular cell type was determined using ELDA webtool at http://bioinf.wehi.edu.au/software/elda.

### SCID mice xenografts

The research protocol was approved by Wayne State University and the VA Medical Center Institutional Animal Care and Use Committees. Four-week-old female ICR/severe combined immunodeficient mice (SCID), obtained from Taconic Laboratory (Germantown, NY) were used for these studies.

To determine the tumorigenic potential of miR-145 overexpressing HCT-116 cells, SCID mice were subcutaneously (s.c.) injected with ∼ 2.5 × 10^5^ HCT-116 cells that were stably transfected with pCMV/miR-145 or pCMV (control).

To further determine whether and to what extent forced expression of miR145 or down-regulation of miR-21 by anti-miR21 would affect the tumorigenic potential of colon cancer cells, SCID mice were subcutaneously injected either with ~ 2.5 × 10^5^ CR HCT-116 or CR HT-29 cells suspended in 100 μl Matrigel. Once palpable tumors were formed, SCID mice bearing xenografts were treated with PEI/miRNA-145, PEI/anti-miRNA-21 complexes or PEI/control once a week by i.p. injection of 0.45 nmol (6 μg) for 3 weeks [24]. Tumor measurements were carried out at multiple time points during the experimental period. The experiment was repeated 3 times with 3–5 mice in each group. Mice were weighed regularly to monitor their well being, and the tumor volumes were estimated as follows: volume (mm^3^) = (*L* × W^2^) / 2, where L and W are the tumor length and width (in mm), respectively.

### Statistical analysis

Unless otherwise stated, data are expressed as mean ± SEM. Wherever applicable, the results were analyzed using analysis of variance followed by Fisher protected least significant differences or Scheffé test. *p* < 0.05 was designated as the level of significance.

## Abbreviations

CRC: colorectal cancer
CSCs/CSLCs: cancer stem or stem-like cells
5-FU: 5-Fluorouracil
Ox: Oxaliplatin
FU-Ox: 5-FU and Oxaliplatin
CR: chemo-resistant
CK-20: cytokeratin-20
RREB1: Ras-responsive element-binding protein 1
ELDA: Extreme limiting dilution analysis
qRT-PCR: quantitative reverse transcription-polymerase chain reaction
PEI: Polyethylenimines
